# Supplementary material for: Facility readiness for decentralized Package of Essential Noncommunicable Disease Interventions-Plus (PEN-Plus) care in nine lower-income countries
Source: PLOS Glob Public Health. 2025 Apr 24;5(4):e0004398. doi: 10.1371/journal.pgph.0004398 (PMC12021207; doi:10.1371/journal.pgph.0004398)
Supplement: S1 Text — (DOCX) [file pgph.0004398.s001.docx]

**Collaborators**

The PEN-Plus Partnership study group includes key stakeholders and contributing researchers from 10 countries initiating PEN-Plus strategy. These individuals contributed to all sections of this research and are listed by country: Abhijit Gadewar, Sunil Jadhao, Yogeshwar Kalkonde, Chetanya Malik (Chhattisgarh, India); Alma Adler, Chantelle Boudreaux, Lauren Brown, Gene Bukhman, Matthew M. Coates, Wubaye Walelgne Dagnaw, Laura Drown, Susan Donnellan, Gina Ferrari, Apoorva Gomber, Rachel Gasana, Paula B Byron, Neil Gupta, Yogesh Jain, Katia Domingues, Sheila Klassen, Gene Kwan, Andrew Marx, Ryan McBain, Amy McLaughlin, Emmanuel Mensah, Gedeon Ngoga, Maia Olsen, Catherine Player, Ramon Ruiz, Devashri Salvi, Ada Thapa, Celina Trujillo, Leslie Wentworth, Emily B. Wroe, Emily Yale, Colin Pfaff, Maryam Mansoor (Center for Integration Science, NCDI Poverty Network Co-Secretariat); Michael Abiyu, Lemma Ayele, Wondu Bekele, Zelalem Mengistu, Temesgen Sileshi, Natnael Alemayehu, Natnael A. Abebe (Ethiopia); Dawson Calixte, Darius Fenelon, Nancy Larco (Haiti); Catherine Karekezi, Gideon Ayodo, Gladwell Gathecha, Peter Mokaya, Lilian Mbau, Zipporah Ali, Yvette Kisaka (Kenya); Sterman Toussaint (Liberia); Jones Masiye, Todd Ruderman, Chiyembekezo Kachimanga, Evelyn Chibwe, Noel Kasomekera (Malawi); Ana Mocumbi, Emílio Tostão, Beatriz Manuel, Yolanda Sabino, Neusa Bay, Sam Patel, Humberto Muquingue, Luisa Panguene, Sergio Chicumbe, Nicole M Salipa, Riaze Rafik, Lucy Ramirez, Edi Fulai, Basilio Cumbane, Lucy Ramirez, Andrea Atzori, Fabio Manenti, Giovanni Putoto, Giacomo Marro (Mozambique); Shiva Adhikary, Krishna Aryal, Phanindra Baral, Meghnath Dhimal, Biraj Karmacharya, Bhagawan Koirala, Sandeepa Karki, Abha Shrestha, Archana Shrestha, Abhinav Vaidhya, Bishwash Maharjan(Nepal); Symaque Dusabeyezu, Innocent Kamali, (Rwanda); Remy Bitwayiki Nkwiro, Marta Patiño, Ian Wurie, Santigie Sesay (Sierra Leone); Mary Mayige, Reuben Mutagaywa, Peter Karoli, Esther Mtumbuka, Julie Makani (Tanzania); Isaac Ssinabulya, Martha Nabadda,Ann R. Akiteng, Robert Kalyesubula, Frank Mugabe, Sarah Asio Eragu. Cinderella Muhangi Ngonzi(Uganda); Roma Chilengi, Fastone Mathew Goma, Mukobe Chisunka, Namasiku Siyumbwa, Wibroad Mutale, Bavin Mulenga(Zambia); Kudakwashe Madzeke, Alexio Mangwiro, Alaisa Mbiriri, Alvern Mutengerere, Wencelas Nyamayaro, Porika Nyawai, Abaden Svisva, Laura Ruckstuhl, Lucia Gonzalez, Justice Mudhavanhu, and Lee Nkala (Zimbabwe). All authors approved the final manuscript and are solely responsible for its accuracy.
