# Supplementary material for: Facility readiness for decentralized Package of Essential Noncommunicable Disease Interventions-Plus (PEN-Plus) care in nine lower-income countries
Source: PLOS Glob Public Health. 2025 Apr 24;5(4):e0004398. doi: 10.1371/journal.pgph.0004398 (PMC12021207; doi:10.1371/journal.pgph.0004398)
Supplement: S1 Data — (PDF) [file pgph.0004398.s002.pdf]

Record ID	4	7	8	15	16	17	18	19	20	22	23	24	25	26	27	28	29	30
Catchment area type (select all that apply) (choice=Urban/Peri-urban)	Checked	Unchecked	Unchecked	Unchecked	Checked	Checked	Unchecked	Unchecked	Checked	Unchecked	Checked	Unchecked	Unchecked	Checked	Checked	Checked	Unchecked	Checked
Catchment area type (select all that apply) (choice=Rural)	Checked	Checked	Checked	Checked	Checked	Checked	Checked	Checked	Checked	Unchecked	Checked	Checked	Checked	Checked	Checked	Unchecked	Checked	Checked
Catchment area type (select all that apply) (choice=Other)	Checked	Unchecked	Unchecked	Unchecked	Unchecked	Unchecked	Unchecked	Unchecked	Checked	Unchecked	Unchecked	Unchecked	Unchecked	Unchecked	Unchecked	Unchecked	Unchecked	Unchecked
Does your facility have electricity from any source (e.g., electricity grid, generator, solar, or other)?		Yes	Yes	Yes	Yes	Yes	No	Yes	Yes	Yes	Yes	Yes	Yes	Yes	Yes	Yes	Yes	Yes
What is the facility's main source of energy? (choice=Central supply of energy (e.g., national or community grid))	Checked	Checked	Checked	Checked	Checked	Checked	Unchecked	Checked	Checked	Checked	Checked	Checked	Unchecked	Checked	Checked	Checked	Checked	Checked
What is the facility's main source of energy? (choice=Generator (fuel or battery operated))	Checked	Checked	Checked	Unchecked	Checked	Checked	Checked	Checked	Checked	Unchecked	Unchecked	Unchecked	Unchecked	Checked	Checked	Checked	Unchecked	Unchecked
What is the facility's main source of energy? (choice=Solar system)	Unchecked	Checked	Checked	Unchecked	Unchecked	Unchecked	Checked	Unchecked	Unchecked	Unchecked	Unchecked	Unchecked	Checked	Unchecked	Checked	Unchecked	Unchecked	Unchecked
What is the facility's main source of energy? (choice=Other)	Unchecked	Unchecked	Unchecked	Unchecked	Unchecked	Unchecked	Unchecked	Unchecked	Unchecked	Unchecked	Unchecked	Unchecked	Unchecked	Unchecked	Unchecked	Unchecked	Unchecked	Unchecked
Please specify.																		
During the past 7 days, was electricity available at all times from the main or any backup source when the facility was open for services?	Always available (no interruptions)	Often available (interruptions of less than 2 hours per	Often available (interruptions of less than 2 hours	Often available (interruptions of less than 2 hours per	Always available (no interruptions)	Always available (no interruptions)	Never available	Always available (no interruptions)	Always available (no interruptions)	Often available (interruptions of less than 2 hours	Always available (no interruptions)	Always available (no interruptions)	Always available (no interruptions)	Always available (no interruptions)	Always available (no interruptions)	Always available (no interruptions)	Always available (no interruptions)	Always available (no interruptions)
Is there access to internet within the facility today?	Yes	Yes	Yes	Yes	Yes	Yes	No	Yes	Yes	Yes	No	Yes	Yes	Yes	Yes	Yes	Yes	Yes
Are there functioning computers at this facility?	Yes	Yes	Yes	Yes	Yes	Yes	No	Yes	Yes	Yes	No	Yes	Yes	Yes	Yes	Yes	Yes	Yes
Does this facility have a functioning telephone that is available to call outside at all times that client services are offered?	Yes	Yes	No	Yes	Yes	Yes	No	Yes	Yes	Yes	No	Yes	Yes	Yes	Yes	Yes	Yes	Yes
Does this facility have a functional ambulance or other vehicle for emergency transportation for clients that is stationed at this facility or operates from this facility?	Yes	Yes	Yes	Yes	Yes	Yes	Yes	Yes	Yes	Yes	No	Yes	Yes	Yes	Yes	Yes	No	No
If no, does this facility have access to an ambulance or other vehicle for emergency transport for clients that is stationed at another facility or that operates from another facility in near proximity?											Yes						Yes	Yes
Is water available on facility premises?	Yes	Yes	Yes	Yes	Yes	Yes	Yes	Yes	Yes	Yes	Yes	Yes	Yes	Yes	Yes	Yes	Yes	Yes
Does this facility have a pharmacy on site?	Yes	Yes	Yes	Yes	Yes	Yes	Yes	Yes	Yes	Yes	Yes	Yes	Yes	Yes	Yes	Yes	Yes	Yes
Lab diagnostics and Equipment																		
Is radiography (x-ray) available at this facility?	Yes	Yes	No	Yes	Yes	Yes	No	Yes	Yes	No	No	Yes	Yes	Yes	Yes	Yes	No	Yes
Is this equipment/test functional today?	Yes	Yes		Yes	Yes	Yes		Yes	Yes			Yes	Yes	Yes	Yes	Yes		Yes
Are blood pressure measuring devices available at this facility?	Yes	Yes	Yes	Yes	Yes	Yes	Yes	Yes	Yes	Yes	Yes	Yes	Yes	Yes	Yes	Yes	Yes	Yes
Is this equipment/test functional today?	Yes	Yes	Yes	Yes	Yes	Yes	Yes	Yes	Yes	Yes	No	Yes	Yes	Yes	Yes	Yes	Yes	Yes
Is a weight scale available at this facility?	Yes	Yes	Yes	Yes	Yes	Yes	Yes	Yes	Yes	Yes	Yes	Yes	Yes	Yes	Yes	Yes	Yes	Yes
Is this equipment/test functional today?	Yes	Yes	Yes	Yes	Yes	Yes	Yes	Yes	Yes	Yes	Yes	Yes	Yes	Yes	Yes	Yes	Yes	Yes
Is a measuring tape or ruler available at this facility?	Yes	Yes	Yes	Yes	Yes		Yes	Yes	Yes	Yes	Yes	Yes	Yes	Yes	Yes		Yes	No
Is this equipment/test functional today?	Yes	Yes	Yes	Yes	Yes		Yes	Yes	Yes	Yes	No	Yes	Yes	Yes	Yes		Yes	
Is hemoglobin testing available at this facility?	Yes	Yes	Yes	Yes	Yes	Yes	Yes	Yes	Yes	Yes	Yes	Yes	Yes	Yes	Yes	Yes	Yes	Yes
Is this equipment/test functional today?	Yes	Yes	Yes	No	Yes	Yes	Yes	No	Yes	Yes	Yes	Yes	Yes	Yes	Yes	Yes	Yes	Yes
Is erythrocyte sedimentation rate testing available at this facility?	Yes	Yes	Yes	Yes	Yes	Yes	No	No	Yes	Yes	No	No	No	Yes	Yes	Yes	Yes	Yes
Is this equipment/test functional today?	Yes	Yes	Yes	Yes	Yes	Yes			Yes	Yes				Yes	Yes	Yes		No
Is glucose testing available at this facility?	Yes	Yes	Yes	Yes	Yes	Yes	Yes	Yes	Yes	Yes	No	Yes	Yes	Yes	Yes	Yes	Yes	Yes
Is this equipment/test functional today?	Yes	Yes	Yes	Yes	Yes	Yes	Yes	Yes	Yes	Yes		Yes	No	Yes	Yes	Yes	Yes	Yes
Is serum electrolyte testing available at this facility?	No	Yes	Yes	No	Yes	Yes	No	No	Yes	Yes	No	Yes	Yes	Yes	Yes		Yes	Yes
Is this equipment/test functional today?		Yes	Yes		No	No			Yes	No		Yes	No	Yes	Yes		Yes	No
Is creatinine testing available at this facility?	Yes	Yes	Yes	Yes	Yes	Yes	No	Yes	Yes	Yes	No	Yes	Yes	Yes	Yes	Yes	Yes	Yes
Is this equipment/test functional today?	Yes	No	Yes	No	No	Yes		Yes	Yes	No		Yes	No	Yes	Yes	Yes	No	No
Is coagulation (prothrombin time (PT)/INR) testing available at this facility?	No	No	Yes	Yes	No	No	No	Yes	No	No	No	No	No	Yes	Yes	No	Yes	No
Is this equipment/test functional today?			Yes	Yes				No						Yes	Yes			
Is blood glucose testing available at this facility?	Yes	Yes	Yes	Yes	Yes	Yes	Yes	Yes	Yes	Yes	Yes	Yes	Yes	Yes	Yes	Yes	Yes	Yes
Is this equipment/test functional today?	Yes	Yes	Yes	No	Yes	Yes	Yes	Yes	Yes	Yes	No	Yes	No	Yes	Yes	Yes	Yes	Yes
Are home glucometers available at this facility?	Yes	Yes	Yes		No	No	No	Yes	Yes			No	Yes	Yes	Yes	No	Yes	Yes
Is this equipment/test functional today?	Yes	Yes	Yes					Yes	Yes				No	Yes	Yes		Yes	Yes
Is monofilament testing available at this facility?	No	No	No	No	No	No	No	No	No		No	No	No	No	No	No	No	No
Is this equipment/test functional today?																		
Is hemoglobin A1c testing available at this facility?	Yes	No	No	No	No	No	No	No	Yes	No	No	Yes	Yes	Yes	Yes	No	Yes	No
Is this equipment/test functional today?	No								No			Yes	No	Yes	Yes			
Is C-peptide testing available at this facility?	No	No	No	No	No	No	No	No	No		No	No	No	No	No	No		No
Is this equipment/test functional today?																		
Is urine ketone testing available at this facility?	Yes	No	Yes		Yes	Yes	Yes	Yes	Yes			Yes	Yes	Yes	Yes	Yes	Yes	Yes
Is this equipment/test functional today?	Yes		Yes		Yes	Yes	Yes	Yes	Yes			Yes	Yes	Yes	Yes	Yes	Yes	Yes
Is electrocardiography available at this facility?	Yes	No	No	Yes	Yes	Yes	No	No	Yes		No	No	No	Yes	Yes	No	No	Yes
Is this equipment/test functional today?	Yes			Yes	No	Yes			Yes					Yes	Yes			No
Is ultrasound equipment with cardiac probes available at this facility?	Yes	No	No	No	No	No	No	No	Yes		No	No	No	Yes	No	Yes	No	No
Is this equipment/test functional today?	No								Yes					Yes		No		
Is ultrasound equipment with abdominal probes available at this facility?	Yes	Yes	Yes	Yes	Yes	Yes	Yes	Yes	Yes		No	No	No	Yes	Yes	Yes	Yes	Yes
Is this equipment/test functional today?	Yes	Yes	Yes	Yes	Yes	No	Yes	Yes	Yes					Yes	Yes	Yes	Yes	Yes
Are liver function tests available at this facility?	Yes	Yes	Yes	Yes	Yes	Yes	No	Yes	Yes	Yes	No	No	No	Yes	Yes		Yes	
Is this equipment/test functional today?	Yes	Yes	Yes	No	No	Yes		Yes	Yes	Yes				Yes	Yes		Yes	
Is hepatitis B and C testing available at this facility?	Yes	Yes	No	Yes	Yes	Yes	Yes	Yes	Yes	Yes	Yes	Yes	Yes	Yes	Yes	Yes	Yes	Yes
Is this equipment/test functional today?	Yes	Yes		No	Yes	No	Yes	Yes	Yes	Yes	No	Yes	Yes	Yes	Yes	No	Yes	Yes
Is hemoglobin testing available at this facility?	Yes	Yes	Yes	Yes	Yes	Yes	Yes	Yes	Yes	Yes	Yes	Yes	Yes	Yes	Yes	Yes	Yes	Yes
Is this equipment/test functional today?	Yes	Yes	Yes	No	Yes	Yes	Yes	Yes	Yes	Yes	No	Yes	Yes	Yes	Yes	Yes	Yes	Yes
Is blood smear available at this facility?	Yes	Yes	Yes	No	Yes	Yes	Yes	Yes	Yes	Yes	Yes	No	No	Yes	Yes	Yes	Yes	Yes
Is this equipment/test functional today?	Yes	Yes	Yes		Yes	Yes	No	Yes	Yes	Yes	No			Yes	Yes	Yes	Yes	No
Is full blood count available at this facility?	Yes	Yes	Yes	Yes	Yes	Yes	No	Yes	Yes	Yes	Yes	Yes	Yes	Yes	Yes	Yes	Yes	Yes
Is this equipment/test functional today?	Yes	No	Yes	No	Yes	Yes		Yes	Yes	Yes	Yes	Yes	Yes	Yes	Yes	Yes	Yes	Yes

Record ID	4	7	8	15	16	17	18	19	20	22	23	24	25	26	27	28	29	30
Are peak flow meters available at this facility?	Yes	No	No	Yes	No	No	No	No	No	Yes	No	No	No	No	Yes	No	No	No
Is this equipment/test functional today?	No			Yes						Yes					Yes			
Are inhalers available at this facility?	No	No	No	Yes	No	No	Yes	Yes	Yes	Yes	No	Yes	Yes	Yes	Yes	No	No	Yes
Is this equipment/test functional today?				No			Yes	Yes	Yes	Yes		Yes	No	Yes	Yes			Yes
Are spacers available at this facility?	No	No	No	No	No	No	No	No	No	Yes	No	No	No	Yes	Yes	No	No	No
Is this equipment/test functional today?										No				Yes	Yes			
Is spirometry available at this facility?	Yes	No	No	No	No	No	No	No	Yes	Yes	No	No	No	Yes	No	No	No	No
Is this equipment/test functional today?	No								Yes	No				Yes				
Are nebulizers available at this facility?	Yes	Yes	Yes	Yes	No	No	Yes	Yes	Yes	Yes	No	Yes	Yes	Yes	Yes	Yes	No	No
Is this equipment/test functional today?	Yes	Yes	Yes	Yes			Yes	Yes	Yes	Yes		Yes	No	Yes	Yes	Yes		
Are nasogastric tubes available at this facility?	Yes	Yes	Yes	Yes	Yes	Yes	Yes	No	Yes	Yes	No	Yes	Yes	Yes	Yes	Yes	Yes	Yes
Is this equipment/test functional today?	Yes	Yes	Yes	Yes	Yes	Yes	Yes		Yes	No		Yes	Yes	Yes	Yes	Yes	Yes	Yes
Are bladder catheters available at this facility?	Yes	Yes	No	Yes	No	Yes	Yes	Yes	Yes	Yes	Yes	Yes	Yes	Yes	Yes		Yes	Yes
Is this equipment/test functional today?	Yes	Yes		Yes		Yes	Yes	Yes	Yes	Yes	Yes	Yes	Yes	Yes	Yes		Yes	No
Is an opioid lock box available at this facility?	No	Yes	No	No	No	Yes	No	No	No	No	No	Yes	Yes	Yes	No	Yes		Yes
Is this equipment/test functional today?		Yes				Yes						Yes	No	Yes		Yes		Yes
Are pressure-reducing mats available at this facility?	No	No	No	No	No	No	No	No	No	No	No	No	No	No	No	No	No	No
Is this equipment/test functional today?																		
Medication																		
Is benzathine penicillin generally available at this facility?	No	Yes	Yes	Yes	Yes	Yes	No	Yes	Yes	Yes	No	No	No	No	No	Yes	Yes	Yes
Is this medication currently stocked?		Yes	Yes	Yes	Yes	Yes		No	Yes	Yes						Yes	Yes	Yes
In the last 3 months, was benzathine penicillin:	Never available	Mostly available	Mostly available	Always available	Always available	Always available	Mostly available	Mostly available	Always available			Rarely available	Rarely available	Never available	Never available	Always available	Always available	Mostly available
Is penicillin V potassium (VK) generally available at this facility?	No	Yes	No	Yes	No	Yes	Yes	Yes	No	Yes	No	No	No	No	No	No	Yes	No
Is this medication currently stocked?		Yes		Yes		Yes	Yes	No		No							Yes	
In the last 3 months, was penicillin V potassium (VK):	Never available	Mostly available	Rarely available	Always available	Never available	Always available	Always available	Always available	Never available			Rarely available	Rarely available	Never available	Never available	Rarely available	Always available	Never available
Is short-acting insulin generally available at this facility?	Yes	Yes	No	Yes	Yes	Yes	No	Yes	Yes	Yes	Yes	Yes	Yes	Yes	Yes	No	Yes	Yes
Is this medication currently stocked?	No	Yes		Yes	Yes	Yes		No	Yes	Yes	Yes	Yes	Yes	Yes	Yes		Yes	Yes
In the last 3 months, was short-acting insulin:	Mostly available	Mostly available	Rarely available	Always available	Always available	Always available	Never available	Always available	Always available			Always available	Always available	Mostly available	Always available	Rarely available	Always available	Always available
Is intermediate-acting insulin generally available at this facility?	Yes	Yes	No	No	Yes	Yes	No	Yes	Yes	Yes	No	Yes	Yes	Yes	Yes	No	Yes	Yes
Is this medication currently stocked?	No	Yes			Yes	Yes		No	Yes	Yes		Yes	Yes	Yes	Yes		Yes	No
In the last 3 months, was intermediate-acting insulin:	Mostly available	Mostly available	Rarely available	Rarely available	Always available	Always available	Never available	Always available	Always available			Mostly available	Mostly available	Mostly available	Always available	Never available	Always available	Always available
Is long-acting insulin generally available at this facility?	No	No	No	No	Yes	Yes	No	No	No	Yes	No	No	No	Yes	Yes	Yes	Yes	Yes
Is this medication currently stocked?					Yes	No				No				Yes	Yes	No	Yes	No
In the last 3 months, was long-acting insulin:	Never available	Never available	Rarely available	Rarely available	Always available	Mostly available	Never available	Always available	Never available			Rarely available	Rarely available	Mostly available	Mostly available	Rarely available	Always available	Always available
Is metformin generally available at this facility?	Yes	Yes	Yes	Yes	Yes	Yes	Yes	Yes	Yes	Yes	No	Yes	Yes	Yes	Yes	Yes	Yes	Yes
Is this medication currently stocked?	Yes	Yes	Yes	Yes	Yes	Yes	Yes	No	Yes	Yes		Yes	Yes	Yes	Yes	Yes	Yes	Yes
In the last 3 months, was metformin:	Always available	Mostly available	Mostly available	Always available	Always available	Always available	Always available	Always available	Always available			Mostly available	Mostly available	Always available	Always available	Rarely available	Always available	Always available
Are sulfonylureas generally available at this facility?	Yes	Yes	Yes	Yes	Yes	Yes	Yes	Yes	Yes	Yes	No	Yes	Yes	Yes	No	Yes	Yes	Yes
Is this medication currently stocked?	Yes	Yes	Yes	Yes	Yes	Yes	Yes	No	Yes	Yes		Yes	Yes	Yes		Yes	Yes	Yes
In the last 3 months, were sulfonylureas:	Always available	Mostly available	Mostly available	Always available	Always available	Always available	Always available	Always available	Always available			Always available	Always available	Always available	Never available	Rarely available	Always available	Always available
Is aspirin generally available at this facility?	Yes	Yes	Yes	Yes	Yes	Yes	Yes	Yes	Yes	Yes	Yes	No	No	Yes	Yes	Yes	No	No
Is this medication currently stocked?	Yes	Yes	Yes	Yes	Yes	Yes	Yes	No	Yes	Yes	Yes			Yes	Yes	Yes		
In the last 3 months, was aspirin:	Always available	Mostly available	Always available	Always available	Always available	Always available	Always available	Always available	Always available			Never available	Rarely available	Always available	Always available		Rarely available	Never available
Are loop diuretics (e.g., furosemide) generally available at this facility?	Yes	Yes	Yes	Yes	Yes	Yes	Yes	Yes	Yes	Yes	Yes	Yes	Yes	Yes	Yes	Yes	Yes	Yes
Is this medication currently stocked?	Yes	Yes	Yes	Yes	Yes	Yes	Yes	No	Yes	Yes	Yes	Yes	Yes	Yes	Yes	Yes	Yes	Yes
In the last 3 months, were loop diuretics:	Mostly available	Mostly available	Mostly available	Always available	Always available	Always available	Always available	Always available	Always available			Mostly available	Mostly available	Always available	Always available	Always available	Always available	Always available
Are angiotensin-converting enzyme (ACE) inhibitors generally available at this facility?	Yes	Yes	Yes	Yes	Yes	Yes	Yes	Yes	Yes	Yes	Yes	Yes	Yes	Yes	Yes	Yes	No	Yes
Is this medication currently stocked?	Yes	Yes	Yes	Yes	Yes	Yes	Yes	No	Yes	Yes	Yes	Yes	Yes	Yes	Yes	Yes		Yes
In the last 3 months, were ACE inhibitors:	Always available	Mostly available	Mostly available	Always available	Always available		Always available	Always available	Always available			Mostly available	Mostly available	Always available	Always available	Mostly available	Rarely available	Mostly available
Are beta-blockers generally available at this facility?	Yes	Yes	Yes	Yes	Yes	No	Yes	Yes	Yes	Yes	No	No	No	Yes	Yes	Yes	Yes	Yes
Is this medication currently stocked?	Yes	Yes	Yes	Yes	Yes		Yes	No	Yes	No				Yes	Yes	Yes	Yes	Yes
In the last 3 months, were beta-blockers:	Always available	Mostly available	Always available	Rarely available	Always available	Mostly available	Always available	Always available	Always available			Rarely available	Rarely available	Always available	Always available		Always available	Always available
Are potassium-sparing diuretics (e.g., spironolactone) generally available at this facility?	No	No	Yes	Yes	Yes	Yes	Yes	Yes	Yes	Yes	No	No	No	Yes	Yes	No	No	No
Is this medication currently stocked?			Yes	Yes	Yes	Yes	Yes	No	Yes	Yes				Yes	Yes			
In the last 3 months, were potassium-sparing diuretics:	Never available	Never available	Always available	Always available	Always available	Always available	Always available	Always available	Always available			Rarely available	Rarely available	Always available	Always available	Never available	Never available	Rarely available
Are thiazide diuretics generally available at this facility?	Yes	Yes	No		Yes	Yes	Yes	Yes	Yes	Yes	Yes	Yes	Yes	Yes	Yes		No	Yes
Is this medication currently stocked?	Yes	Yes			Yes	Yes	Yes	No	Yes	Yes	No	Yes	Yes	Yes	No			No
In the last 3 months, were thiazide diuretics:	Always available	Mostly available	Never available		Always available	Always available	Always available	Always available	Always available			Always available	Always available	Always available	Mostly available		Rarely available	Always available
Are calcium channel blockers generally available at this facility?	Yes	Yes	No	Yes	Yes	Yes	Yes	Yes	Yes	Yes	No	Yes	Yes	Yes	Yes	Yes	No	Yes
Is this medication currently stocked?	Yes	Yes		Yes	Yes	Yes	Yes	No	Yes	Yes		Yes	Yes	Yes	Yes	No		Yes
In the last 3 months, were calcium channel blockers:	Always available	Mostly available	Rarely available	Rarely available	Always available		Always available	Always available	Always available			Mostly available	Mostly available	Always available	Mostly available	Mostly available	Rarely available	Always available
Is methyldopa generally available at this facility?	Yes	Yes	No	Yes	Yes	Yes	Yes	Yes	Yes	Yes	No	Yes	Yes	Yes	Yes	Yes	Yes	Yes
Is this medication currently stocked?	Yes	Yes		Yes	Yes	Yes	Yes	No	Yes	No		Yes	Yes	Yes	Yes	No	Yes	No
In the last 3 months, was methyldopa:	Mostly available	Mostly available	Rarely available	Always available	Mostly available	Always available	Always available		Rarely available			Always available	Always available	Rarely available	Rarely available	Rarely available	Always available	Always available
Are nitrates generally available at this facility?	No	No	No	No	No	No	No	Yes	Yes	Yes	No	No	No	Yes	Yes	Yes	No	Yes
Is this medication currently stocked?								No	Yes	No				Yes	No	Yes		No
In the last 3 months, were nitrates:	Never available	Never available	Rarely available	Never available	Never available	Never available			Mostly available			Never available	Rarely available	Always available	Rarely available	Mostly available		Always available
Is heparin generally available at this facility?	Yes	No	No	No	No	No	No	Yes	Yes	Yes	No	No	No	No	Yes	Yes	No	No
Is this medication currently stocked?	No							No	Yes	No					Yes	Yes		
In the last 3 months, was heparin:	Mostly available	Never available	Rarely available	Never available	Never available	Never available	Never available		Always available			Never available	Rarely available	Never available	Mostly available	Mostly available		Rarely available
Is warfarin generally available at this facility?	No	No	Yes	No	No	No	No	Yes	No	Yes	No	No	No	Yes	Yes	Yes	No	No
Is this medication currently stocked?			Yes					No		Yes				Yes	Yes	No		
In the last 3 months, was warfarin:	Never available	Never available	Always available	Never available	Never available	Never available	Never available		Never available			Never available	Rarely available	Always available	Always available	Never available	Rarely available	Never available
Are other (non-warfarin) oral anti-coagulants (e.g., enoxaparin, rivaroxaban) generally available at this facility?	Yes	No	No	No	No	No	No	Yes	Yes	No	No	No	No	No	Yes	No	No	No

Record ID	4	7	8	15	16	17	18	19	20	22	23	24	25	26	27	28	29	30
Are these medications currently stocked?	No							No	Yes						Yes			
In the last 3 months, were other oral anti-coagulants:	Rarely available	Never available	Rarely available	Never available	Never available	Never available			Always available			Never available	Never available	Never available	Always available	Never available	Rarely available	Never available
Is potassium (oral) generally available at this facility?	Yes	No	No	No	Yes	Yes	No	No	Yes	No	No	No	No	No	No	Yes	Yes	No
Is this medication currently stocked?	No				No	Yes			Yes							No	No	
In the last 3 months, was potassium (oral):	Mostly available	Never available	Rarely available	Never available	Rarely available	Always available			Mostly available			Never available	Never available	Never available	Always available	Mostly available	Rarely available	Never available
Are proton-pump inhibitors generally available at this facility?	Yes	Yes	Yes	Yes	Yes	Yes	Yes	Yes	Yes	Yes	Yes	No	No	Yes	Yes	Yes	No	Yes
Is this medication currently stocked?	Yes	Yes	Yes	Yes	Yes	Yes	Yes	No	Yes	Yes	Yes			Yes	Yes	No		Yes
In the last 3 months, were proton-pump inhibitors:	Always available	Mostly available	Always available	Always available	Always available	Always available	Always available	Always available	Always available			Rarely available	Rarely available	Always available	Always available	Rarely available		Always available
Is lactulose generally available at this facility?	Yes	No	Yes	No	No	No	Yes	Yes	Yes	Yes	No	No	No	Yes	Yes	No	No	No
Is this medication currently stocked?	Yes		Yes				Yes	Yes	Yes	Yes				Yes	Yes			
In the last 3 months, was lactulose:	Always available	Never available	Mostly available	Never available	Never available	Never available	Always available	Always available	Always available			Never available	Never available	Always available	Always available	Never available	Rarely available	Rarely available
Are statins generally available at this facility?	Yes	No	Yes	No	No	Yes	Yes	Yes	Yes	Yes	No	No	No	Yes	No	Yes	No	Yes
Is this medication currently stocked?	Yes		Yes			Yes	Yes	Yes	Yes	No				Yes		No		Yes
In the last 3 months, were statins:	Always available	Never available	Mostly available	Rarely available	Never available	Always available	Always available	Always available	Always available			Never available	Never available	Always available	Rarely available	Rarely available	Rarely available	Always available
Is hydralazine generally available at this facility?	No	Yes	Yes	Yes	Yes	Yes	No	Yes	No	Yes	No	Yes	Yes	Yes	No	Yes	Yes	Yes
Is this medication currently stocked?		Yes	Yes	Yes	Yes	Yes		Yes		No		Yes	Yes	Yes		Yes	Yes	Yes
In the last 3 months, was hydralazine:	Never available	Mostly available	Mostly available	Always available	Always available	Always available	Never available	Always available	Never available			Mostly available	Mostly available	Always available	Rarely available	Mostly available	Always available	Always available
Is isosorbide dinitrate generally available at this facility?	Yes	No	No	Yes	No	No	No	No	Yes	No	No	No	No	No	Yes		No	Yes
Is this medication currently stocked?	Yes			Yes					Yes						No			Yes
In the last 3 months, was isosorbide dinitrate:	Always available	Never available	Rarely available	Rarely available	Never available	Never available	Never available		Always available			Never available	Never available	Never available	Rarely available		Rarely available	Always available
Is hydroxyurea generally available at this facility?	Yes	No	Yes	No	No	No	No	Yes	Yes	No	No	No	No	No	Yes			No
Is this medication currently stocked?	Yes		Yes					No	Yes						Yes			
In the last 3 months, was hydroxyurea:	Mostly available	Never available	Always available	Never available	Never available	Never available	Never available		Always available			Never available	Never available	Rarely available	Always available			Never available
Are prophylactic antibiotics generally available at this facility?	Yes	Yes	Yes	Yes	Yes	Yes	Yes	Yes	Yes	No	No	Yes	Yes	Yes	Yes	No	Yes	No
Is this medication currently stocked?	Yes	Yes	Yes	Yes	Yes	Yes	Yes	No	Yes			Yes	Yes	Yes	Yes		Yes	
In the last 3 months, were prophylactic antibiotics:	Always available	Always available	Always available	Always available	Mostly available	Always available	Always available		Always available			Mostly available	Mostly available	Always available	Always available	Rarely available	Always available	Never available
Are inhaled corticosteroids generally available at this facility?	No	No	Yes	Yes	Yes	Yes	No	Yes	Yes	Yes	No	No	No	Yes	Yes	No	No	No
Is this medication currently stocked?			Yes	Yes	No	No		No	Yes	No				Yes	Yes			
In the last 3 months, were inhaled corticosteroids:	Never available	Never available	Always available	Always available	Mostly available	Always available	Never available	Always available	Always available			Rarely available	Rarely available	Always available	Always available	Never available	Never available	Never available
Is aminophylline generally available at this facility?	Yes	Yes	No	Yes	Yes	Yes	Yes	No	Yes	Yes	No	No	No	No	No		Yes	No
Is this medication currently stocked?	No	Yes		Yes	Yes	Yes	Yes		Yes	No							Yes	
In the last 3 months, was aminophylline:	Rarely available	Mostly available	Rarely available	Always available	Always available	Always available	Always available	Always available	Always available			Rarely available	Rarely available	Never available	Rarely available		Always available	Never available
Are oral corticosteroids generally available at this facility?	Yes	Yes	Yes	Yes	Yes	Yes	Yes	Yes	Yes	Yes	No	Yes	No	Yes	No		Yes	Yes
Is this medication currently stocked?	No	Yes	Yes	Yes	Yes	No	Yes	No	Yes	No		No		Yes			Yes	Yes
In the last 3 months, were oral corticosteroids:	Mostly available	Mostly available	Always available	Always available	Always available	Rarely available	Rarely available	Always available	Always available			Mostly available	Mostly available	Always available	Never available		Always available	Always available
Is oral morphine or other opioids generally available at this facility?	No	Yes	Yes	Yes	Yes	Yes	No	Yes	No	No	No	No	No	Yes	Yes	No	No	Yes
Is this medication currently stocked?		Yes	Yes	Yes	Yes	Yes		No						Yes	No			Yes
In the last 3 months, was oral morphine or other opioids:	Never available	Mostly available	Always available	Always available	Always available	Always available	Rarely available		Never available			Rarely available	Rarely available	Rarely available	Always available	Never available	Never available	Always available
Are anti-emetics generally available at this facility?	Yes	Yes	No	Yes	Yes	Yes	Yes	Yes	Yes	Yes	Yes	Yes	Yes	Yes	Yes		Yes	Yes
Is this medication currently stocked?	Yes	Yes		Yes	Yes	Yes	Yes	No	Yes	Yes	Yes	Yes	Yes	Yes	Yes		Yes	No
In the last 3 months, were anti-emetics:	Always available	Mostly available	Rarely available	Always available	Always available	Rarely available	Always available	Always available	Always available			Mostly available	Mostly available	Always available	Always available		Never available	Always available
Are anti-depressants generally available at this facility?	Yes	Yes	Yes	Yes	Yes	Yes	No	Yes	Yes	Yes	No	Yes	Yes	Yes	Yes	Yes	Yes	Yes
Is this medication currently stocked?	Yes	Yes	Yes	Yes	Yes	No		No	Yes	Yes		Yes	Yes	Yes	Yes	Yes	Yes	Yes
In the last 3 months, were anti-depressants:	Always available	Mostly available	Mostly available	Always available	Always available	Rarely available			Always available			Always available	Always available	Always available	Always available	Mostly available	Always available	Always available
Are anti-psychotics generally available at this facility?	Yes	Yes	Yes	Yes	Yes	Yes	No	Yes	Yes	Yes	No	Yes	Yes	Yes	Yes	Yes	Yes	Yes
Is this medication currently stocked?	Yes	Yes	Yes	Yes	Yes	Yes		No	Yes	Yes		No	Yes	Yes	Yes	Yes	Yes	No
In the last 3 months, were anti-psychotics:	Always available	Mostly available	Mostly available	Always available	Always available	Always available	Never available		Always available			Mostly available	Mostly available	Always available	Always available	Mostly available	Always available	Always available
Are laxatives generally available at this facility?	Yes	Yes	No	Yes	Yes	Yes	Yes	Yes	Yes	Yes	Yes	No	No	Yes	Yes		No	Yes
Is this medication currently stocked?	Yes	Yes		Yes	Yes	Yes	Yes	No	Yes	Yes	Yes			Yes	Yes			Yes
In the last 3 months, were laxatives:	Always available	Mostly available	Rarely available	Always available	Always available	Always available	Always available	Always available	Always available			Rarely available	Rarely available	Always available	Always available	Rarely available		Always available
Is paracetamol generally available at this facility?	Yes	Yes	Yes	Yes	Yes	Yes	Yes	Yes	Yes	Yes	No	Yes	Yes	Yes	Yes	Yes	Yes	Yes
Is this medication currently stocked?	Yes	Yes	Yes	Yes	Yes	Yes	Yes	No	Yes	Yes		Yes	Yes	Yes	Yes	Yes	Yes	Yes
In the last 3 months, was paracetamol:	Always available	Mostly available	Mostly available	Mostly available	Always available	Rarely available	Always available	Always available	Always available			Always available	Mostly available	Always available	Always available	Mostly available	Always available	Always available
Is topical antifungal generally available at this facility?	Yes	Yes	Yes		Yes	Yes	Yes	Yes	Yes	Yes	No	No	No	Yes	Yes	No	Yes	Yes
Is this medication currently stocked?	Yes	Yes	Yes		Yes	Yes	Yes	No	Yes	Yes				Yes	Yes		Yes	No
In the last 3 months, was topical antifungal:	Always available	Mostly available	Always available		Always available	Always available	Always available	Always available	Always available			Never available	Rarely available	Always available	Always available	Never available	Always available	Always available
Is ibuprofen generally available at this facility?	Yes	Yes	No	Yes	Yes	Yes	Yes	Yes	Yes	Yes	Yes	Yes	Yes	Yes	Yes	Yes	Yes	Yes
In the last 3 months, was ibuprofen:	Always available	Mostly available	Rarely available	Rarely available	Always available	Always available	Always available	Always available	Always available			Mostly available	Mostly available	Always available	Always available	Rarely available	Mostly available	Rarely available
