# Supplementary material for: Facility readiness for decentralized Package of Essential Noncommunicable Disease Interventions-Plus (PEN-Plus) care in nine lower-income countries
Source: PLOS Glob Public Health. 2025 Apr 24;5(4):e0004398. doi: 10.1371/journal.pgph.0004398 (PMC12021207; doi:10.1371/journal.pgph.0004398)
Supplement: S1 Appendix — (PDF) [file pgph.0004398.s003.pdf]

PEN-Plus Partnership Baseline Survey
Part A: Facility Overview

Instructions: Please complete the following questionnaire with regards to the entire health facility, consulting the Medical Director and/or other personnel as needed. Input from multiple personnel may be needed to complete questionnaire sections and relevant individuals should be consulted if necessary.

Facility Background

1. Name of facility: ____________________

2. County: ____________________

3. Region/province: ____________________

4. District: ____________________

5. Facility location
   ☐ Urban/Peri-urban
   ☐ Rural
   ☐ Other (specify): ____________________

6. Catchment area population: __________________

7. Catchment area type (select all that apply)
   ☐ Urban/Peri-urban
   ☐ Rural
   ☐ Other (specify): ____________________

8. Managing authority
   ☐ Government/public
   ☐ Non-governmental organization (NGO)/Not-for-profit
   ☐ Private-for-profit
   ☐ Mission/faith-based
   ☐ Other (specify): ____________________

9. Please describe any implementing partners that are working to support activities at this facility.

 ____
 ____
 ____

10. Does this facility currently receive any additional, direct, non-financial forms of support (e.g., supplies and equipment, training, mentorship that are provided directly to the facility and staff)?

 ☐ Yes

 Please describe this support.

 ____
 ____

 ☐ No

11. Please indicate the time period that is used for routine reporting to the government on the number of outpatients visits at this facility (e.g., monthly, quarterly, etc).

 ____

12. How many outpatient visits were conducted at this facility during the last reporting period?

 ____

13. Please indicate the number of inpatient beds currently in place at this facility.

 Total: _____
 Adult: _____
 Pediatric: _____
 Intensive care unit (ICU) or high-dependency unit (HDU): _____
 Other (specify: ____________): _____
 Other (specify: ____________): _____
 Other (specify: ____________): _____

14. On average, how many hours per day is this facility open for patients to present for care?

 ☐ 8 hours
 ☐ 12 hours
 ☐ 24 hours
 ☐ Other (specify): __________

15. Does this facility offer after hours and weekend outpatient care?

- ☐ After-hours only
- ☐ Weekend only
- ☐ After-hours and weekend care
- ☐ Neither

16. Please describe service lines/departments present at this facility according to the table below.

**What are the service lines/departments in this facility? (List all)**	**What cadres of health workers work there?**	**How many health workers are on a shift at the same time in this service line/department?**	**What conditions are managed in each service line/department?**
EXAMPLE:<br><br>*Operating room*	*General surgeon*<br>*Nurse anesthetist*<br>*Surgical nurse*	*6*	*Appendicitis*<br>*Obstructed labor*<br>*Open fracture*<br>*Hernia*

e.g., medical outpatient department (OPD), noncommunicable disease (NCD) clinic, antenatal care (ANC) clinic, pediatric ward

Condition	Can this condition be managed at this facility?	If yes -					
		**What service line/unit/team typically manages these in the ambulatory/out patient setting**	**Which health worker categories are involved in management of this condition?**	**Which health worker category is in charge of overseeing management?**	**Please estimate how many patients are currently in care.**	**What are the sources of data you use for this estimation?**	**Optional: Please use registers/ logbooks to determine the number of patients in care in the last reporting period.**
Rheumatic heart disease	Y / N						
Heart failure	Y / N						
Congenital heart disease	Y / N						
Hypertension	Y / N						
Stroke	Y / N						
Type 1 diabetes	Y / N						

Type 2 diabetes	Y / N						
Sickle cell disease	Y / N						
Chronic kidney disease	Y / N						
Chronic respiratory disease	Y / N						
Chronic liver disease	Y / N						
Epilepsy	Y / N						
Chronic cancers, including breast cancer and chronic myeloid leukemia	Y / N						
Palliative care, including for advanced malignancies	Y / N						

Staffing

1. Please indicate actual numbers of staff of each type are currently employed at this facility.				
**Category**	**Employed full-time**	**Employed part-time**	**In this position for <u>at least 6 months</u>**	**Are these staff visiting or permanent?**
Generalist (non-specialist) physician				
Specialist physician (overall)				
Non-physician clinicians/paramedical professionals (e.g. clinical officers, medical assistants)				
Nursing professionals				
Specialist nurses				
Social worker				

**Specialist**	**Is this specialist type employed at the clinic where outpatient services for severe NCDs are provided?**	**If no, is this specialist type employed elsewhere at this facility?**	**Are these specialists visiting or permanent?**
Endocrinologist	Y / N	Y / N / NA	
Pediatric endocrinologist	Y / N	Y / N / NA	
Cardiologist	Y / N	Y / N / NA	
Pulmonologist	Y / N	Y / N / NA	
Nephrologist	Y / N	Y / N / NA	
Gastroenterologist	Y / N	Y / N / NA	
General surgeon	Y / N	Y / N / NA	
Hepatologist	Y / N	Y / N / NA	
Hematologist	Y / N	Y / N / NA	
Oncologist	Y / N	Y / N / NA	
Radiologist	Y / N	Y / N / NA	
Rheumatologist	Y / N	Y / N / NA	
Psychiatrist	Y / N	Y / N / NA	
Other (specify):__________	Y / N	Y / N / NA	
Other (specify):__________	Y / N	Y / N / NA	

2. Please identify any sub-specialist providers available **at the clinic where outpatient services for severe NCDs are provided.**

____
____

3. Please identify any sub-specialist providers available **elsewhere at this facility.**<br>____<br>____

Infrastructure

Communications	
1. Does this facility have a functioning telephone that is available to call outside at all times that client services are offered?	Y / N
2. Is there access to internet within the facility today?	Y / N
4. Are there functioning computers at this facility?	Y / N
Ambulance/transport for emergencies	
5. Does this facility have a functional ambulance or other vehicle for emergency transportation for clients that is stationed at this facility or operates from this facility?<br>a. If no, does this facility have access to an ambulance or other vehicle for emergency transport for clients that is stationed at another facility or that operates from another facility in near proximity?<br>i. If yes, how far from the facility is this vehicle? ________________________	Y / N<br><br>Y / N
6. Is fuel for the ambulance or other emergency vehicle available today?	Y / N
Power supply	
7. Does your facility have electricity from any source (e.g., electricity grid, generator, solar, or other)?<br>a. If yes, what is the facility's main source of energy?<br>☐ Central supply of energy (e.g., national or community grid)<br>☐ Generator (fuel or battery operated)<br>☐ Solar system<br>☐ Other ________________________________	Y / N
8. During the past 7 days, was electricity available at all times from the main or any backup source when the facility was open for services?<br>☐ Always available (no interruptions)<br>☐ Often available (interruptions of less than 2 hours per day)<br>☐ Sometimes available (frequent or prolonged interruptions of more than 2 hours per day)<br>☐ Rarely available (intermittently or infrequently available)<br>☐ Never available	
Basic client amenities	

9. Is water available on facility premises?	Y / N
10. What is the most commonly used source of water for the facility at this time?<br>☐ Piped into facility<br>☐ Piped onto facility grounds<br>☐ Public tap/standpipe<br>☐ Tubewell/borehole<br>☐ Protected dug well<br>☐ Unprotected dug well<br>☐ Protected spring<br>☐ Unprotected spring<br>☐ Rainwater collection<br>☐ Bottled water<br>☐ Cart w/ small tank/drum<br>☐ Tanker truck<br>☐ Surface water<br>☐ Other (specify): ___________________________<br>☐ No water source	
11. Does this facility have a pharmacy on site?	Y / N

Financing

User Fees	
1. Please indicate whether patients pay fees for the following:	
Consultation	Y / N<br>If yes, indicate the average fee: ________
Laboratory tests	Y / N<br>If yes, indicate the average fee: ________
X-rays and other imaging	Y / N<br>If yes, indicate the average fee: ________
Supplies (e.g., compresses, syringes, etc.)	Y / N<br>If yes, indicate the average fee: ________
Medicines	Y / N<br>If yes, indicate the average fee: ________
If available, please attach a copy of the fee schedule when you return this questionnaire	
2. Are any patients exempt from paying user fees?<br>a. If yes, please specify the circumstances.<br>____<br>_____________________	Y / N
Insurance	
3. Does this facility participate in a health insurance scheme?<br>a. If yes, please indicate the types of insurance schemes accepted.<br>☐ Public<br>☐ Private<br>☐ Community-based	Y / N

Diagnostics, Medications, & Equipment

1. Who is the principal person responsible for managing the ordering of medications and medical supplies at this facility?
   - ☐ Nurse
   - ☐ Clinical officer
   - ☐ Pharmacy technician
   - ☐ Pharmacy assistant
   - ☐ Pharmacist
   - ☐ Medical assistant
   - ☐ Other (specify): ____

2. Which of the following mechanisms is used to determine this facility's resupply quantities?
   - ☐ The facility itself (pull distribution system)

     Please indicate which individual at the facility is responsible for determining what is needed and quantities.

     ____

     How are needed materials and quantities determined?

     ____

     ____
   - ☐ A higher-level facility (push distribution system)
   - ☐ Other (specify): ____

3. What is the main source of your routine pharmaceutical commodity supplies?
   - ☐ National medical stores
   - ☐ Joint medical stores
   - ☐ NGO/Donors
   - ☐ Private sources
   - ☐ Other (specify):

     ____

4. Does this facility have a reliable method of storing medicine refrigerated between 2 and 8 degrees Celsius?
   - ☐ Yes
   - ☐ No

Lab, diagnostics, and equipment

5. Availability of lab, diagnostics, and equipment			
**Disease system (or category)**	**Equipment/test**	**Please indicate if equipment/test is…**	
		Available at facility	*Functional today (if available)*
Multi-specialty			
	Radiography (X-ray)	Y / N	Y / N / NA
	Blood pressure measuring devices	Y / N	Y / N / NA
	Weight scale	Y / N	Y / N / NA
	Measuring tape or ruler	Y / N	Y / N / NA
	Hemoglobin	Y / N	Y / N / NA
	Erythrocyte sedimentation rate	Y / N	Y / N / NA
	Glucose	Y / N	Y / N / NA
	Serum electrolytes	Y / N	Y / N / NA
	Creatinine	Y / N	Y / N / NA
	Coagulation (prothrombin time (PT)/INR)	Y / N	Y / N / NA
Endocrine			
	Blood glucose	Y / N	Y / N / NA
	Home glucometers	Y / N	Y / N / NA
	Monofilament	Y / N	Y / N / NA
	Hemoglobin A1c	Y / N	Y / N / NA
	C-peptide	Y / N	Y / N / NA
	Urine ketone testing	Y / N	Y / N / NA
Cardiovascular, renal, and hepatology			
	Electrocardiography	Y / N	Y / N / NA
	Ultrasound equipment with cardiac probes	Y / N	Y / N / NA
	Ultrasound equipment with abdominal probes	Y / N	Y / N / NA
	Liver function tests	Y / N	Y / N / NA
	Hepatitis B and C testing	Y / N	Y / N / NA
Hematology			
	Hemoglobin	Y / N	Y / N / NA
	Blood smear	Y / N	Y / N / NA
	Full blood count	Y / N	Y / N / NA
Pulmonology			
	Peak flow meters	Y / N	Y / N / NA
	Inhalers	Y / N	Y / N / NA
	Spacers	Y / N	Y / N / NA
	Spirometry	Y / N	Y / N / NA
	Nebulizers	Y / N	Y / N / NA

Palliative care		
Nasogastric tube	Y / N	Y / N / NA
Bladder catheter	Y / N	Y / N / NA
Opioid lock box	Y / N	Y / N / NA
Pressure-reducing mat	Y / N	Y / N / NA
Palliative care		
Nasogastric tube	Y / N	Y / N / NA
Bladder catheter	Y / N	Y / N / NA
Opioid lock box	Y / N	Y / N / NA
Pressure-reducing mat	Y / N	Y / N / NA
Other (add as needed)		
	Y / N	Y / N / NA
	Y / N	Y / N / NA
	Y / N	Y / N / NA
	Y / N	Y / N / NA
	Y / N	Y / N / NA
	Y / N	Y / N / NA

Medications

6. Availability of medications				
**Disease system (or category)**	**Medication**	**Please indicate if medication is…** *Generally available at facility*	*Currently stocked*	**In the last 3 months, was this medication:**
Multi-specialty				
	Benzathine penicillin	Y / N	Y / N / NA	☐ Always available ☐ Mostly available ☐ Rarely available ☐ Never available
	Penicillin V Potassium (VK)	Y / N	Y / N / NA	☐ Always available ☐ Mostly available ☐ Rarely available ☐ Never available
Endocrine				
	Short-acting insulin	Y / N	Y / N / NA	☐ Always available ☐ Mostly available ☐ Rarely available ☐ Never available
	Intermediate-acting insulin	Y / N	Y / N / NA	☐ Always available ☐ Mostly available ☐ Rarely available ☐ Never available
	Long-acting insulin	Y / N	Y / N / NA	☐ Always available ☐ Mostly available ☐ Rarely available ☐ Never available
	Metformin	Y / N	Y / N / NA	☐ Always available ☐ Mostly available ☐ Rarely available ☐ Never available
	Sulfonylureas	Y / N	Y / N / NA	☐ Always available ☐ Mostly available ☐ Rarely available ☐ Never available
Cardiovascular, renal, and hepatology				
	Aspirin	Y / N	Y / N / NA	☐ Always available ☐ Mostly available ☐ Rarely available ☐ Never available
	Loop diuretics (e.g. furosemide)	Y / N	Y / N / NA	☐ Always available ☐ Mostly available ☐ Rarely available ☐ Never available
	Angiotensin-converting enzyme (ACE) inhibitors	Y / N	Y / N / NA	☐ Always available ☐ Mostly available

			☐ Rarely available ☐ Never available
Beta-blockers	Y / N	Y / N / NA	☐ Always available ☐ Mostly available<br>☐ Rarely available ☐ Never available
Potassium-sparing diuretic (e.g. spironolactone)	Y / N	Y / N / NA	☐ Always available ☐ Mostly available<br>☐ Rarely available ☐ Never available
Thiazide diuretics	Y / N	Y / N / NA	☐ Always available ☐ Mostly available<br>☐ Rarely available ☐ Never available
Calcium channel blockers	Y / N	Y / N / NA	☐ Always available ☐ Mostly available<br>☐ Rarely available ☐ Never available
Methyldopa	Y / N	Y / N / NA	☐ Always available ☐ Mostly available<br>☐ Rarely available ☐ Never available
Nitrates	Y / N	Y / N / NA	☐ Always available ☐ Mostly available<br>☐ Rarely available ☐ Never available
Heparin	Y / N	Y / N / NA	☐ Always available ☐ Mostly available<br>☐ Rarely available ☐ Never available
Warfarin	Y / N	Y / N / NA	☐ Always available ☐ Mostly available<br>☐ Rarely available ☐ Never available
Other (non-warfarin) oral anti-coagulant (e.g. enoxaparin, rivaroxaban)	Y / N	Y / N / NA	☐ Always available ☐ Mostly available<br>☐ Rarely available ☐ Never available
Potassium, oral	Y / N	Y / N / NA	☐ Always available ☐ Mostly available<br>☐ Rarely available ☐ Never available
Proton-pump inhibitors	Y / N	Y / N / NA	☐ Always available ☐ Mostly available<br>☐ Rarely available ☐ Never available
Lactulose	Y / N	Y / N / NA	☐ Always available ☐ Mostly available<br>☐ Rarely available ☐ Never available
Statins	Y / N	Y / N / NA	☐ Always available ☐ Mostly available<br>☐ Rarely available ☐ Never available

Hydralazine	Y / N	Y / N / NA	☐ Always available ☐ Mostly available ☐ Rarely available ☐ Never available
Isosorbide dinitrate	Y / N	Y / N / NA	☐ Always available ☐ Mostly available ☐ Rarely available ☐ Never available
Hematology			
Hydroxyurea	Y / N	Y / N / NA	☐ Always available ☐ Mostly available ☐ Rarely available ☐ Never available
Prophylactic antibiotics	Y / N	Y / N / NA	☐ Always available ☐ Mostly available ☐ Rarely available ☐ Never available
Pulmonary			
Inhaled corticosteroids	Y / N	Y / N / NA	☐ Always available ☐ Mostly available ☐ Rarely available ☐ Never available
Aminophylline	Y / N	Y / N / NA	☐ Always available ☐ Mostly available ☐ Rarely available ☐ Never available
Oral corticosteroids	Y / N	Y / N / NA	☐ Always available ☐ Mostly available ☐ Rarely available ☐ Never available
Palliative care			
Oral morphine or other opioids	Y / N	Y / N / NA	☐ Always available ☐ Mostly available ☐ Rarely available ☐ Never available
Anti-emetics	Y / N	Y / N / NA	☐ Always available ☐ Mostly available ☐ Rarely available ☐ Never available
Anti-depressants	Y / N	Y / N / NA	☐ Always available ☐ Mostly available ☐ Rarely available ☐ Never available
Anti-psychotics	Y / N	Y / N / NA	☐ Always available ☐ Mostly available ☐ Rarely available ☐ Never available
Laxatives	Y / N	Y / N / NA	☐ Always available ☐ Mostly available ☐ Rarely available ☐ Never available
Paracetamol	Y / N	Y / N / NA	☐ Always available ☐ Mostly available

			☐ Rarely available ☐ Never available
Topical antifungal	Y / N	Y / N / NA	☐ Always available ☐ Mostly available<br>☐ Rarely available ☐ Never available
Ibuprofen	Y / N	Y / N / NA	☐ Always available ☐ Mostly available<br>☐ Rarely available ☐ Never available
Other (add as needed)			
	Y / N	Y / N / NA	☐ Always available ☐ Mostly available<br>☐ Rarely available ☐ Never available
	Y / N	Y / N / NA	☐ Always available ☐ Mostly available<br>☐ Rarely available ☐ Never available
	Y / N	Y / N / NA	☐ Always available ☐ Mostly available<br>☐ Rarely available ☐ Never available
	Y / N	Y / N / NA	☐ Always available ☐ Mostly available<br>☐ Rarely available ☐ Never available
	Y / N	Y / N / NA	☐ Always available ☐ Mostly available<br>☐ Rarely available ☐ Never available
	Y / N	Y / N / NA	☐ Always available ☐ Mostly available<br>☐ Rarely available ☐ Never available

PEN-Plus Partnership Baseline Survey
Part B: Non-Communicable Disease (NCD) Management and Health Information/Data Systems

Instructions: Please complete the following questionnaire with regards to the unit which is responsible for providing the majority of outpatient services for severe non-communicable diseases (NCDs) (e.g., insulin-dependent diabetes, cardiovascular conditions, sickle cell disease, chronic kidney disease, stroke, etc) at this facility. If these services occur in multiple places, please complete these questions for the location that is responsible for the majority of services. Input from multiple personnel may be needed to complete questionnaire sections and relevant individuals should be consulted if necessary.

NCD Management

1. What is the name of this unit?

   ____

2. Please use the following options to describe this clinic.

   ☐ Specialized clinic
   ☐ General outpatient department (OPD) with specialty teams or sub-clinics (specify):
   ____
   ☐ General OPD without specialty teams or sub-clinics
   ☐ Other (specify):
   ____

3. Are NCD patients seen in a designated physical space?

   ☐ Yes
   ☐ No

4. Is there a room with auditory and visual privacy available for NCD patient consultations?

   ☐ Auditory privacy only
   ☐ Visual privacy only
   ☐ Both auditory and visual privacy
   ☐ No privacy

5. What NCD conditions are managed here (select all that apply)?
   ☐ Rheumatic heart disease
   ☐ Heart failure
   ☐ Congenital heart disease
   ☐ Severe hypertension
   ☐ Stroke
   ☐ Type 1 diabetes
   ☐ Type 2 diabetes
   ☐ Sickle cell disease
   ☐ Chronic kidney disease (CKD)
   ☐ Chronic respiratory disease
   ☐ Chronic liver disease
   ☐ Epilepsy
   ☐ Chronic cancers, including breast cancer and chronic myeloid leukemia (CML)
   ☐ Palliative care, including for advanced malignancies

6. When is this clinic open?
   ☐ Every day
   ☐ Select days (specify): ________________________
   ☐ As facility decides; no set schedule

7. How is the clinic staffed (select all that apply)?
   ☐ The same staff work in the clinic continually
   ☐ Facility staff rotate through the clinic
   ☐ Other (specify): _________________________

8. Are patients formally enrolled or registered into care in this clinic?
   ☐ Yes
   ☐ No

9. Do NCD patients attend routine follow-up visits at this clinic?
   ☐ Yes
   ☐ No

10. Do patients receive follow up appointment dates?
   ☐ Yes
   ☐ No

11. Does this facility have an established definition or criteria for NCD patients considered lost to follow-up?

    ☐ Yes

    a. Please describe:
    ____
    ____
    ____

    ☐ No

12. Is there a system in place at this facility to identify patients who have missed follow-up visits or are considered lost to follow-up?

    ☐ Yes

    a. Please describe:
    ____
    ____
    ____

    ☐ No

13. Is there a system in place at this facility to contact patients who have missed follow-up visits or are considered lost to follow-up? Y/N

    ☐ Yes

    a. Please describe:
    ____
    ____
    ____

    ☐ No

14. What is the <u>primary</u> receiving facility for NCD patients referred out of this facility for a higher level of care?

    ____

15. How long is the typical travel time to reach the receiving facility from this facility?

    _____ min

16. If an <u>established</u> NCD patient arrives at the facility in crisis, is this clinical team informed?

☐ Yes

a. How? ____

b. Are patient records transferred to this clinical team?

☐ Yes

☐ No

☐ No

17. Are inpatients with newly identified NCD conditions transferred to this clinic for further care?

☐ Yes

a. Are patient records transferred to this clinical team?

☐ Yes

☐ No

☐ No

18. Does this facility receive patients with NCDs referred from other health facilities?

☐ Yes

a. Which facilities? __________________

b. Do you receive medical records for transferred patients?

☐ Yes

☐ No

☐ No

19. Does this facility receive NCD-related referrals from screening and/or surveillance activities conducted in the community?

☐ Yes

☐ No

20. Please list all guidelines or clinical aids used at this clinic to manage NCD conditions.

____

____

____

21. Are NCD-related educational materials available to patients at this facility?

☐ Yes

a. Please list all NCD-related materials available to patients.

____
____
____
____

☐ No

22. Are NCD-related community education materials available at this facility?

☐ Yes

a. Please list all NCD-related community education materials.

____
____
____
____

☐ No

23. Is social support available to NCD patients at this facility?

☐ Yes

a. Please describe social support activities currently available.

____
____
____
____

☐ No

24. Provision of NCD-related activities				
		**If activity is provided…**	**If activity is <u>not</u> provided in this clinic…**	
**Activity**	**Is this activity currently available at this clinic?**	**Who (cadre) provides this activity?**	**Is this activity provided elsewhere at this facility? If yes, please specify department/unit.**	**Are patients requiring this activity referred to a different facility?**
Cardiac disease				
Diagnosis				
Ultrasonography with cardiac probes	Y / N		Y / N ____________	Y / N
Ultrasonography with abdominal probes	Y / N		Y / N ____________	Y / N
Management				
Beta blocker management	Y / N		Y / N ____________	Y / N
Warfarin or other anticoagulation management	Y / N		Y / N ____________	Y / N
International normalized ratio (INR) testing	Y / N		Y / N ____________	Y / N

Diuretic management	Y / N		Y / N<br>____________________	Y / N
Angiotensin-converting enzyme (ACE) inhibitor management	Y / N		Y / N<br>____________________	Y / N
Surgical referral	Y / N		Y / N<br>____________________	Y / N
Severe hypertension				
Diagnosis				
Hypertension screening	Y / N		Y / N<br>____________________	Y / N
Management				
Medical management with anti-hypertensives in 3 or more classes	Y / N		Y / N<br>____________________	Y / N
Stroke				
Diagnosis				
Imaging for diagnosis (computed tomography (CT) or magnetic resonance imaging (MRI))	Y / N		Y / N<br>____________________	Y / N
Management				
Aspirin management	Y / N		Y / N<br>____________________	Y / N
Anti-hypertensive management	Y / N		Y / N<br>____________________	Y / N
Physical therapy	Y/N		Y / N<br>____________________	Y / N
Type 1 diabetes				
Diagnosis				
Urine glucose testing	Y / N		Y / N<br>____________________	Y / N

Blood glucose testing	Y / N		Y / N<br>________________	Y / N
Hemoglobin A1c testing	Y / N		Y / N<br>________________	Y / N
C-peptide testing	Y / N		Y / N<br>________________	Y / N
Urine ketone testing	Y / N		Y / N<br>________________	Y / N
Management				
Insulin management	Y / N		Y / N<br>________________	Y / N
Metformin management	Y / N		Y / N<br>________________	Y / N
Sulfonylurea management	Y / N		Y / N<br>________________	Y / N
Retinal screening	Y / N		Y / N<br>________________	Y / N
Monofilament testing	Y / N		Y / N<br>________________	Y / N
CKD screening	Y / N		Y / N<br>________________	Y / N
Type 2 diabetes				
Diagnosis				
Urine glucose testing	Y / N		Y / N<br>________________	Y / N
Blood glucose testing	Y / N		Y / N<br>________________	Y / N
Hemoglobin A1c testing	Y / N		Y / N<br>________________	Y / N
C-peptide testing	Y / N		Y / N<br>________________	Y / N

Management				
Insulin management	Y / N		Y / N<br>______________________	Y / N
Metformin management	Y / N		Y / N<br>______________________	Y / N
Sulfonylurea management	Y / N		Y / N<br>______________________	Y / N
Retinal screening	Y / N		Y / N<br>______________________	Y / N
Monofilament testing	Y / N		Y / N<br>______________________	Y / N
CKD screening	Y / N		Y / N<br>______________________	Y / N
Sickle cell disease				
Diagnosis				
Any diagnostic testing for sickle cell (please specify): ___________________________	Y / N		Y / N<br>______________________	Y / N
Transcranial Doppler ultrasound	Y / N		Y / N<br>______________________	Y / N
Management				
Initiation and monitoring of hydroxyurea	Y / N		Y / N<br>______________________	Y / N
Antibiotic therapy	Y / N		Y / N<br>______________________	Y / N
Pain management, opioids	Y / N		Y / N<br>______________________	Y / N
Blood transfusion	Y / N		Y / N<br>______________________	Y / N
Chronic kidney disease				

Diagnosis				
Urine protein testing	Y / N		Y / N<br>________________	Y / N
Serum creatinine testing	Y / N		Y / N<br>________________	Y / N
Management				
Diuretic management	Y / N		Y / N<br>________________	Y / N
Antihypertensive management	Y / N		Y / N<br>________________	Y / N
Chronic respiratory disease				
Diagnosis				
Spirometry	Y / N		Y / N<br>________________	Y / N
Chest X-ray	Y / N		Y / N<br>________________	Y / N
Management				
Bronchodilator and steroid inhaler management	Y / N		Y / N<br>________________	Y / N
Steroid inhaler management	Y / N		Y / N<br>________________	Y / N
Oral steroid management	Y / N		Y / N<br>________________	Y / N
Chronic liver disease				
Diagnosis				
Abdominal ultrasound	Y / N		Y / N<br>________________	Y / N
Liver enzyme testing	Y / N		Y / N<br>________________	Y / N
Platelet testing	Y / N		Y / N	Y / N

			____________________	
Management				
Medication management for cirrhosis	Y / N		Y / N ____________________	Y / N
Hepatitis C treatment	Y / N		Y / N ____________________	Y / N
Hepatitis B treatment	Y / N		Y / N ____________________	Y / N
Diuretic management (e.g., furosemide, spironolactone)	Y / N		Y / N ____________________	Y / N
Propranolol management	Y / N		Y / N ____________________	Y / N
Lactulose management	Y / N		Y / N ____________________	Y / N
Epilepsy				
Management				
Anti-seizure medication management	Y / N		Y / N ____________________	Y / N
Chronic cancer, including breast cancer and chronic myeloid leukemia				
Management				
Chronic oral therapy (e.g., tamoxifen)	Y / N		Y / N ____________________	Y / N
Palliative care, including for advanced malignancies				
Pain management, opioids	Y / N		Y / N ____________________	Y / N
Pain management, non-opioids	Y / N		Y / N ____________________	Y / N
Other symptom management	Y / N		Y / N ____________________	Y / N

25. Are patients with type 1 diabetes provided with supplies for home management (e.g., home glucometer, testing strips, syringes, ketone strips)?

☐ Yes

a. Please list supplies provided to patients for home use.

____

____

____

☐ No

Health Information and Data Systems

Data Systems

1. Does this facility have a medical record system for NCD patients?

☐ Yes

a. Please indicate the level at which this system is used (select all that apply).

☐ National level

☐ Regional or state level

☐ Facility level

☐ No

b. Please indicate the types of medical record system is used at the following locations.

What type of medical record system is used at the facility as a whole? (Multiple answers possible)	Which medical record system(s) is used in this clinic (where outpatient services for severe NCDs are provided)? (Multiple answers possible)	Which medical record system(s) is used in the **inpatient wards?** (Multiple answers possible)
☐ Facility-level electronic medical record system<br>☐ Clinic-specific electronic medical record system<br>☐ Facility-level paper patient chart (across all service areas)<br>☐ Clinic-specific paper records	☐ Facility-level electronic medical record system<br>☐ Clinic-specific electronic medical record system<br>☐ Facility-level paper patient chart (across all service areas)<br>☐ Clinic-specific paper records	☐ Facility-level electronic medical record system<br>☐ Clinic-specific electronic medical record system<br>☐ Facility-level paper patient chart (across all service areas)<br>☐ Clinic-specific paper records

☐ Clinic logbook or register<br>☐ Patient appointment card (patient keeps)<br>☐ Other (specify):<br>_________________	☐ Clinic logbook or register<br>☐ Patient appointment card (patient keeps)<br>☐ Other (specify):<br>_________________<br>☐ Not applicable	☐ Clinic logbook or register<br>☐ Patient appointment card (patient keeps)<br>☐ Other (specify):<br>_________________

2. If this clinic uses an electronic medical record (EMR) system:

    a. What software is used (e.g., OpenMRS)?
    Note: If different software are used please mention all and specify where they are used.

    ____

    b. Is this system used to collect patient-level medical record information about NCDs?

    ☐ Yes

    a. Do you collect information on patients' diagnosis and clinical measurements (e.g. lab values, symptoms)?

    ☐ Yes
    ☐ No

    b. Do you export data from this software?

    ☐ Yes
    ☐ No

    c. How long has this software been used at this facility?

    ____

    d. Do you need internet to run this software?

    ☐ Yes
    ☐ No

    e. Do you have a stable internet connection at this facility?

    ☐ Yes
    ☐ No

f. What devices do staff members use to run this software? (Select all that apply)

☐ Smart phone
☐ Tablet
☐ Laptop computer
☐ Desktop computer

g. Other comments about the EMR system:

1. What is one thing you like about the electronic system?
____
____

2. What is one thing that could be better?
____
____

☐ No

h. Do you collect any information on patients' diagnosis and clinical measurements (e.g. lab values, symptoms)?

☐ Yes
☐ No

i. How is the data from the paper forms, charts or registers digitized for reporting? (Select all that apply)

☐ Entered into Excel
☐ Entered into cloud-based form (Eg: Google form or Microsoft forms)
☐ DHIS2
☐ Data are stored in physical files/archives
☐ Data is not entered or digitized
☐ Other, please specify___________

j. Please share an example of the paper form(s) used at this clinic as a soft copy (photo or scan)

c. Other comments about the paper medical record system:

i. What is one thing you like about the paper system?

____
____

ii. What is one thing that could be better?

____
____

4. Please describe any clinical registers/logbooks utilized in this clinic.

____
____

5. Please describe any clinical registers/logbooks utilized in inpatient wards where NCD patients receive treatment.

____
____

6. Please describe any routine processes for reporting of aggregated NCD data at this facility (e.g., health management information system (HMIS), programmatic, other).

____
____

Data Completeness

7. Thinking about all the patients seen at this clinic, and all the patients you have data for, please rate the level of data completeness.

☐ New or mostly unused [Generally not used, new or in disuse]
☐ Very incomplete [complete for < 25% of patients]
☐ Moderately complete [complete for 25-75% of patients]
☐ Mostly complete [complete for >75 % of patients]
☐ Nearly or fully complete [nearly 100% complete]

Data Systems Related Staffing

8. Do you have a designated staff member in charge of recording and maintaining data at this clinic?

☐ Yes

a. How long have they been in this role?

_______ years

b. Do they have other clinical duties

☐ Yes

☐ No

c. Have they been trained in data collection practices?

☐ Yes

i. If yes, what training: ___________

☐ No

d. Is this person hired by a specific project?

☐ Only works on specific data

☐ Works on all clinical data

☐ No

e. How much time do clinicians spend in entering data on average in a week?

___________ (hours)

f. Are clinicians trained on data collection at point of care? (Eg: entering patient information on paper or electronic forms, prescriptions)

☐ Yes

i. If yes, what training: ___________

☐ No

Data Use

9. Do you regularly review collected data?

☐ Yes

a. How often does your team review patient related data (patient counts, outcomes etc.)? (Select all that apply)

☐ Weekly
☐ Monthly
☐ Quarterly
☐ Annually
☐ Team does not review data

☐ No

10. Do you use data reports to make decisions about facility or clinical operations?

☐ Yes

a. How do you use this data? (Select all that apply)

☐ Allocation of resources
☐ Patient demographics
☐ Performance review for providers
☐ Setting goals/targets
☐ Seasonal variations in service utilization
☐ Quality improvement
☐ Community mobilization/other activities
☐ Specific analysis
(patient outcomes vs. risk factors or treatment approaches)
☐ Other (specify): __________________

b. Who creates these reports at this clinic? ______________

☐ No

11. Are there any data you wish you could collect? Why?

____
____

Technology

12. Do clinical staff have access to internet in this clinic?

☐ Yes

a. Is this internet reliably available?

☐ Yes

☐ No

b. Does the internet bandwidth support video?

☐ Yes

☐ No

13. Do any programs at the hospital currently use telemedicine?

☐ Yes

☐ No
