# Supplementary material for: Facility readiness for decentralized Package of Essential Noncommunicable Disease Interventions-Plus (PEN-Plus) care in nine lower-income countries
Source: PLOS Glob Public Health. 2025 Apr 24;5(4):e0004398. doi: 10.1371/journal.pgph.0004398 (PMC12021207; doi:10.1371/journal.pgph.0004398)
Supplement: Attachment — Submitted filename: Response to Reviewers.docx [file pgph.0004398.s005.docx]

Dear Editors,

Thank you for the comments on our manuscript. We have now responded to all comments. You can see our responses below.

Alma Adler on behalf of all authors.

**Reviewer #1:**

 The article meets the criteria for PLOS Global public health journal publication and has a sound manuscript with conclusions that are supported by data presented in the results section. The design for the primary research question posed projects an ethically and methodologically sound design with mentioned limitations and weaknesses. This is further elaborated by the drawn conclusions which even with the mentioned limitations in the design, not only answer the question posed but go on to approximate the readiness of 16 centers in 9 low to middle income countries towards 3 severe chronic non communicable diseases.

However, data availability seems inadequate as some of the procedures and surveys implemented are not fully detailed. The manuscript is presented in good palatable english

Some recommendations;

Provide the full meaning of PEN-plus in the title to provide transparency

The full title now reads as follows for clarity:

“Facility readiness for decentralized Package of Essential Noncommunicable Disease Interventions-Plus (PEN-Plus) care in nine lower-income countries”

Line 65- according to WHO (<https://www.who.int/news-room/fact-sheets/detail/noncommunicable-diseases> & <https://www.afro.who.int/health-topics/noncommunicable-diseases>), respiratory conditions, cancers, rheumatic conditions and Hypertension are also conditions we should be ready for so to strengthen your 3 choices you need a substantial literature review supporting why you chose the 3 you chose for the 9 countries selected.

Thank you for this comment. We acknowledge the importance of a broader and diverse range of NCDs, particularly for the population that PEN-Plus seeks to serve. The development and WHO-AFRO adoption of PEN-Plus was built upon a longer effort to document the epidemiology of disease, and NCDIs in particular, among the world’s poorest billion (lines 62-84,1-13). We have referenced the initial work, the 2020 Lancet Commission, which documented the disproportionate burden of severe NCDIs among those in extreme poverty, and particularly among those under 40. We have revised the background to better describe the role that PEN-Plus plays in this broader landscape.

We also cite the work describing the initial development of the PEN-Plus package (rows 77-82, references 5, 7-11). While PEN-Plus is not limited to managing these conditions, we further document the emphasis of these conditions in the programmatic monitoring (rows 82-88, references 5,11-13).

Line 66- grammar error (some text missing)

This has now been corrected.

Line 118/119- survey design is mentioned but not fully described, to support reproducibility of results all data need to be available.

Thank you for your comment. We have modified the Study design section as follows:

“We developed two surveys (Part A and Part B [Appendix A]) as part of this baseline assessment. Part A of the survey consisted of facility-wide assessment, including questions on facility background, infrastructure, service lines/department present at the facility, conditions managed, staffing, diagnostics, medications, and equipment’s. Part B survey was more tailored to NCD specific management and health information data systems. The survey was developed in a collaborative process beginning in November 2021, building on our team’s previous work analyzing equipment and medication availability for NCDs [15, 16]. Survey design was guided by strategic information needed to inform PEN-Plus initiation at the selected clinics. The survey underwent multiple iterative rounds of development where initial draft was created by the research team and clinicians provided input throughout the development process. After refinement of an initial survey, we consulted with partners in each country to ensure the tool was appropriate to the target settings. Once finalized the tool was then distributed to the sites in two formats: electronically through REDCap(17,18) and as a paper- based to accommodate varying levels of technological access.”

We have now included data as supplementary material in Supplementary file 1.

Line 141- provide more details on the survey, the questionnaire, language used, qualifications of trainers, assessments of understanding and what criteria justified clarifying or confirming responses etc-

Thank you. This section has been revised and reads as follows:

“The finalized survey was distributed to all selected sites in March 2022 and sites were given the opportunity to complete the survey via paper forms or electronic entry using REDCap (Research Electronic Data Capture) [17, 18]. Electronic surveys were built in REDCap and members from all country teams were provided with training sessions with research team staff conducted over Zoom. These sessions provided detail walkthrough of the survey structure, the REDCap interface, and instructions for completing the surveys. Virtual office hours were held for the countries to address the question regarding survey completion and data input. Training sessions were provided by the researcher with experience in survey administration and familiar with the PEN-plus initiative. Four countries elected to complete the surveys electronically using REDCap following a virtual training session, while the remaining completed paper forms. Paper surveys were manually entered into REDCap by Boston-based staff. Most countries administered the survey in English as originally written, though one translated it into the local language (Portuguese). The translation process involved collaboration with the bilingual team members familiar with technical terminology used in the survey. We received responses from all facilities over the period of June 2022 to February 2023. Responses represented status of the facilities at the time of survey submission. No human subjects were included in this research.

All surveys were checked for completeness and consistency centrally by the research team. Sites were contacted and resent previous surveys to complete and/or correct questions that were incomplete or unclear in previous responses. In some instances, we asked sites to confirm or clarify responses. This process was repeated as needed until surveys were complete.”

Line 162/163 what time point was the survey conducted because given the time range of june 2022-february 2023, its not clear which time point was the data collected to assess the availability of medicines, equipment etc.

Thank you for this question. Line 172-174 now specifies that responses represented status of the facilities at the time of survey submission. The manuscript previously specified that we assessed availability of medicines, equipment, and supplies at the time of the survey (lines 167-168), as the survey asked specifically about current status of all items.

**Reviewer #2:** 
Topic: Facility readiness for decentralized PEN-Plus care for severe chronic noncommunicable diseases in nine lower-income countries

Summary: The study assessed baseline facility readiness of 16 selected clinics in nine low-income countries as part of a mixed-methods evaluation of the implementation of the PEN-PLUS strategy, which aims to address gaps in access to care for SC-NCDs by integrating and decentralizing care.

Here are a few comments to improve the manuscript:

Abstract
1) Line 47: Not sure whether it is “hospitals” or clinics”, please correct accordingly

Thank you. This has been corrected to facilities.

2) Line 55: The interpretation of index scores (0.75, 0.39, 0.49) is not clear from the abstract. The authors may add an explanation or maybe put in percent.

All index scores have been converted to percentages for improved clarity . Thank you for this suggestion.

Introduction
1) Line 66: cross out “than”

This has now been corrected
2) Line 85: add a comma, before “, nine”
This has now been corrected
Materials and methods
1) Line 163-4; For ease of interpretation, I would suggest converting the index scores to percentages, by multiplying by 100.

As noted above, all index scores have been converted to percentages.
2) Line 175-6: More details on ethics considerations? Was ethics sought in each country? Through what Ethics Committees/Institutions? Anonymity?
This section has been revised and now reads:

“This study was reviewed and determined to be not human subject’s research by the Mass General Brigham IRB. . This work was deemed exempt or covered by Mass General Brigham #2022P001390, Nepal Health Research Council 571/2022 P, National Institute for Medical Research (Tanzania) NIMR/Hq/R.8aVol.IX/4184, University of Zambia Biomedical Research Ethics Committee #3032-2022, Mulago Hospital Research and Ethics Committee MHREC 2022-74, Sierra Leone Ethics and Review Committee SLESRC 027/01/2025, Medical Research Council of Zimbabwe MRCZ/E/347, Amhara Public Health Institute NoH/R/T/T/D/07/43, and Oromia Health Care Bureau BF/HBTFH/H6/2027.”

Results
1) No further comments
Discussion
1) Line 282: cross out the first “in”

This has now been corrected
